# Real-Time Kinetics of Internalization of Anti-EGFR DNA Aptamers and Aptamer Constructs into Cells Derived from Glioblastoma Patients as Indicated by Doxorubicin

**DOI:** 10.3390/ijms26178712

**Published:** 2025-09-07

**Authors:** Valeria Ivko, Olga Antipova, Boris Ivanov, Vadim Tashlitsky, Fatima Dzarieva, Nadezhda Samoylenkova, Dmitry Usachev, Galina Pavlova, Alexey Kopylov

**Affiliations:** 1 Belozersky Research Institute of Physical Chemical Biology, Lomonosov Moscow State University, 119991 Moscow, Russia; valerian.moiseenko@gmail.com (V.I.); antipovachem@gmail.com (O.A.); ivanovb661@yandex.ru (B.I.); tashlitsky@belozersky.msu.ru (V.T.); 2 Department of Chemistry, Lomonosov Moscow State University, 119991 Moscow, Russia; 3 Institution N. N. Burdenko National Medical Research Center of Neurosurgery of the Ministry of Health of the Russian Federation, 125047 Moscow, Russia; dz.fatima@mail.ru (F.D.); samoylenkova.n@gmail.com (N.S.); dousachev@nsi.ru (D.U.); lkorochkin@mail.ru (G.P.); 4 Institute of Higher Nervous Activity and Neurophysiology, Russian Academy of Sciences, 117485 Moscow, Russia

**Keywords:** aptamer, EGFR, doxorubicin, glioblastoma, aptamer constructs, real-time internalization kinetics

## Abstract

The WHO considers the Epidermal Growth Factor Receptor (EGFR) one of the key biomarkers of glioblastoma (GB). EGFR can be identified and targeted using molecular recognition elements (MoREs), like aptamers and aptamer–drug conjugates (ApDCs). Understanding the kinetics of anti-EGFR ApDC interactions with EGFR as well as the kinetics of their internalization into the cells is a crucial step for the further development of anti-EGFR ApDCs. For the first time, a novel approach was implemented to study real-time kinetics by measuring the cellular index (CI) using impedance (xCELLigence). Doxorubicin (DOX) was used as an indicator drug. Because DOX intercalates into the DNA double helix, aptamer–DOX non-covalent complexes were obtained. For the anti-EGFR DNA aptamer GR20, an additional duplex was constructed by synthesizing the extra region (GR20h) and via hybridization with the complementary oligonucleotide (h’) to form a duplex (hh’), thus creating the aptamer construct with complementary oligonucleotide (ACCO) GR20hh’. The original HPLC method quantified the assembly efficiency of an ACCO. The ACCO GR20hh’ retained affinity for the recombinant extracellular domain of EGFR, as measured using Biolayer Interferometry (BLI). According to cytofluorimetry, the ACCO GR20hh’ interacts with cells of continuous culture from GB patient (CCGBP) surgical samples. The DOX–ACCO GR20hh’ complexes are more efficiently internalized by EGFR+ cells lines A-431 and CCGBP 107 than DOX alone.

## 1. Introduction

Glioblastomas (GBs) are aggressive malignant brain tumors with a short median patient survival [1]. Modern protocols for the treatment of glioblastomas include surgical resection, as well as chemo- and radiotherapy [2,3]. The specificity of conventional chemotherapeutic drugs is usually based on the rapid division of tumor cells and their active metabolism (TMZ, etc.). Alternative approaches, such as differentiation therapy [4], have a fundamentally different paradigm.

Tumor cells have specific tumor markers on their surface [5] and, according to the WHO classification, one of the key markers of GB cells is Epidermal Growth Factor Receptor (EGFR) [6,7].

EGFR on tumor cells can be identified using molecular recognition elements (MoREs). The most widely used MoREs are monoclonal antibodies (Cetuximab, Panitumumab, etc.) or their derivatives [7,8]. In addition to antibodies, other peptide-based MoREs (coined as scaffolds) are used, for example, Ankyrin Repeat Proteins (DARPins) [9,10]. Peptides can also be considered as mini-MoREs, but their high conformational mobility does not result in good affinities to targets [11,12,13].

Aptamers are short, single-stranded nucleic acids that bind to proteins or other biomolecules; aptamers are ten-times smaller than antibodies, and they are obtained with chemical synthesis. Aptamers are chemically and structurally stable and easy to renature, store, and apply. The small size of aptamers provides good diffusion and distribution in the body, and it enhances the penetration into tissues and tumors. The non-immunogenic nature of aptamers makes them an excellent therapeutic candidate. To date, there are more than 300 publications devoted to anti-EGFR aptamers. Various RNA and DNA aptamers with different affinities and specificities have been described [14,15,16,17,18].

### 1.1. Covalent MoRE–Drug Conjugates

MoREs have not been very effective receptor blockers, including EGFR, for inhibiting proliferation. Therefore, much attention has been paid to the development of MoREs that are covalently conjugated with cytotoxins, for example, antibody–drug conjugates (ADCs). To further enhance the efficiency of ADCs, peptidase-hydrolysable linkers are used for conjugation, which ensure the rapid release of active molecules [19,20,21,22].

In this work, doxorubicin (DOX) was used as an indicator cytotoxic agent. DOX is a well-known anthracycline antitumor therapeutic drug [23]. The basis of the cellular toxicity of DOX is its intercalation into DNA [24]. According to several in vitro studies, DOX effectively acts on glioma cells [25,26]. Currently, DOX has limited application in medicine because of its cardiotoxicity, which is a serious unwanted side-effect [27]. An example of a MoRE peptide used for glioma treatment is a conjugate of DOX with a natural peptide analogue of chlorotoxin [28].

Similarly to ADCs, the application of aptamer–drug covalent conjugates (ApDCs) has been recently described [29]. Anti-EGFR aptamer E07 [30] that is conjugated with MMAE and MMAF reduced the survival of pancreatic tumor cells with high EGFR abundance. DOX is also used to make ApDCs, for example, with the anti-nucleolin aptamer AS1411 [31]. So far, there are around fifteen publications describing the use of anti-EGFR aptamers and DOX, the majority of which are dedicated to breast cancer. To our knowledge, there are no publications investigating the use of anti-EGFR aptamer–DOX conjugates for the treatment of gliomas. Our group was the first to describe the application of the covalently conjugated DNA aptamer GR20 with DOX on GB cells [32].

### 1.2. Non-Covalent MoRE–DOX Complexes

Apart from the conjugates where DOX is covalently attached to MoREs, the unique chemical structure of DOX allows for the creation of chemically different non-covalent complex MoREs/DOX due to DOX’s ability to intercalate into the DNA double helix. To obtain monoclonal antibody (mAb) complexes with DOX, mAbs are covalently modified with the DNA duplex followed by DOX intercalation into the duplex. For example, Liu et al. demonstrated the selective delivery of DOX by the anti-EGFR mAb-DNA duplex chimera to overexpressing EGFR cells [33].

In aptamers, the existence of hairpin double-stranded regions provides a possibility for DOX intercalation directly into the aptamer. Such non-covalent complexes may be even more therapeutically advantageous than covalent ApDCs since, firstly, there is no DOX inactivation due to chemical modification, and, secondly, they do not hinder DOX release from the ApDCs after internalization into the cell.

To date, there are only few publications describing non-covalent complexes of DOX with aptamers. These complexes can be divided into three groups. The first group includes complexes of DOX with the aptamers themselves, where the function of containers is performed by the double-stranded regions of the aptamers. The second group includes complexes with additional synthetic double-stranded regions that are added to the aptamer as DOX containers. And the third group includes non-covalent aptamer constructions with a complementary oligonucleotide (ACCO). In this case, the original aptamer is synthesized with an additional single-stranded region at the end, which is hybridized with a complementary oligonucleotide. As a result, the complementary double-stranded region becomes a DOX container. To date, there are no data in PubMed (except our first attempt [32]) describing the application of anti-EGFR complexes with either Ap-DOX or ACCO-DOX for glioma treatment. 

DOX appears to be a useful drug for testing the capacities and the kinetics of targeted drug delivery to cells using aptamers, including an ACCO. The affinity of DOX to DNA provides a convenient range of active concentrations for cell studies. Moreover, new technologies for monitoring cell viability in real time, such as impedance measurement (xCELLigence), make it possible to study not only receptor-dependent delivery but also the kinetics of the interaction between the receptors and the complexes. 

Anti-EGFR DNA aptamers are promising MoREs for targeting glioma cells. They are not toxic and, as we demonstrated, can carry toxic loads, like DOX, without losing affinity. In our previous work, we described the DNA aptamer GR20 [34] that is a derivative of the original aptamer U31 [35] selected for EGFR+ cells. Both aptamers demonstrated high affinity for the recombinant extracellular domain of EGFR [34,36]. In the present work, we studied DOX complexes with the anti-EGFR DNA aptamers U31, GR20 and the construct ACCO GR20hh’. DOX intercalates quite well into aptamers, and its binding can be enhanced by creating an additional double-stranded module in the non-covalent construct ACCO GR20hh’. This construct is stable, binds DOX better than the original aptamer and retains affinity for the EGFR target.

Here, we propose a novel approach for real-time analysis of the kinetics of the uptake of DOX–aptamer/ACCO complexes, which is based on measuring the cell index (CI) using the xCELLigence Real-Time Cell Analysis (RTCA). Conventional cell lines A-431, U87, MCF-7, as well as the cells of continuous culture from a GB patient, CCGBP 107, were used for the tests. The ACCO GR20hh’ was internalized by EGFR+ cells more efficiently than DOX alone, which was indicated by a sharp drop in cell index monitoring. This effect was observed for both the conventional overexpressing EGFR+ cell line, A-431, and for the patient cells CCGBP 107.

## 2. Results and Discussion

### 2.1. Models of Putative Secondary Structures of Anti-EGFR DNA Aptamers and ACCO Design for the U31 Family

**Figure 1:**
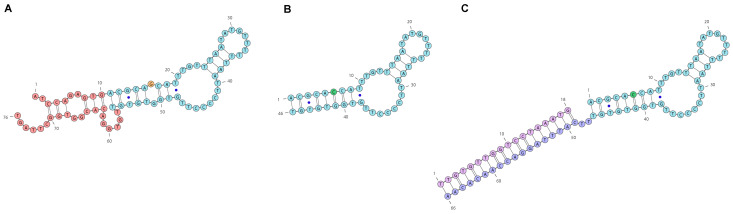
Putative secondary structures of the anti-EGFR DNA aptamers U31 (**A**), GR20 (**B**) and ACCO GR20hh’ (**C**). In the aptamer U31 sequence, the deleted region is shown in red; in addition, 16G (**A**—orange) was replaced by C (**B**—green) to make the aptamer GR20; non-canonical G-T pairs are marked with blue dots.

To engineer the structure of an aptamer, it is required to have a model of a putative secondary structure. Currently, modeling is performed with open-source programs: a popular one is The ViennaRNA Package 2.0 [37,38]. This program provides several options and, therefore, final selection will depend on preferred criteria. In Figure 1, the selected putative secondary structures of the anti-EGFR DNA aptamers are shown. Previously, the modeling of a putative secondary structure of the parent 76-mer aptamer U31 was made [34,36]. Based on this model, a truncated mutant version with a single-nucleotide substitution was obtained, the 46 nt aptamer GR20, which has affinities similar to the parent aptamer U31 [34,36]. The 5′- and 3′-terminal regions (1–10 and 57–76, respectively; U31 numbering) were deleted, removing the terminal’s imperfect hairpin. The G6-G41 opposition in the aptamer U31 was converted into a complementary C6-G41 pair in the aptamer GR20 by a G6C substitution (GR20 numbering).

Based on the minimal putative structure of the aptamer GR20 (46 nt with 4 G-C pairs), the ACCO GR20–GR20hh’ (84 nt, 11 G-C pairs) was created where the deleted terminal’s imperfect duplex of the parental aptamer U31 (3 + 3 pairs, 4 G-C pairs, Figure 1A) was replaced by a perfect duplex (18 pairs, 7 G-C pairs, Figure 1C). The artificial non-covalent ACCO–GR20hh’ has 84 nucleotides with 11 G-C pairs (Figure 1C). It is made via the hybridization of an additional 20 nt region at the 3′-end of the aptamer GR20h (66 nt, 4 G-C pairs) and a complementary 18-mer oligonucleotide h’, having 7 nt Gs + Cs.

Various modifications of the complementary 18-mer oligonucleotides h’, for example, the introduction of a reactive group, a fluorescent label, a drug, surface immobilization, etc., can provide many possibilities for ACCO applications. A similar approach was previously described by Ellington’s laboratory [39,40,41]. There is no universal algorithm for the design of an ACCO duplex structure, since it depends on the specific tasks for an ACCO application. We chose the specific structure of the terminal duplex based on the following considerations. Firstly, the length of the oligonucleotide ‘h’ should ensure duplex stability in the ACCO structure and should not distort the expected secondary structure of the original GR20 aptamer. Secondly, the duplex in the structure should ensure enough intercalation of DOX to inhibit cell growth. All the above parameters are difficult to calculate in advance; therefore, we took an 18-mer duplex with an arbitrary sequence.

#### Proton Magnetic Resonance Spectrometry of the Aptamer GR20

A direct method for detecting the presence of a secondary structure in short aptamers with imperfect hairpins in solution is proton magnetic resonance spectrometry [42,43].

**Figure 2:**
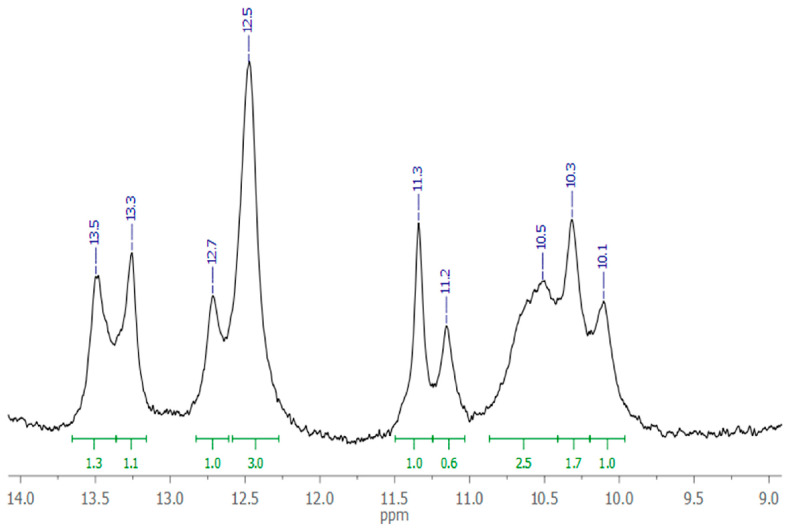
Watson–Crick imino-proton region of ^1^H NMR spectrum of 220 μM aptamer GR20 in PBS with 10% D_2_O, pH 7.0, 4 °C.

The small size of the aptamer GR20 provides the possibility to have it in quantities sufficient for conventional ^1^H NMR measurements. The region of 12.0–13.0 ppm corresponds to imino-protons of G-C pairs, and the region of 9.8–11.6 ppm corresponds to imino-protons of A-T pairs. A ratio of integrated signals in the corresponding regions ranges from 4:7 to 6:5, indicating the presence of a secondary structure of the aptamer at 4 °C (Figure 2).

The intensity ratio of the integrated signals is slightly different from the ratio of A-T and G-C pairs in the proposed secondary structure model (7:4). However, given the lower thermal stability of A-T pairs, it is understandable that the experimental ratio does not fit exactly. More detailed information on the aptamer structure is not required now, since the cost of establishing it does not justify the information gained.

### 2.2. Assembly of the Aptamer Construct with the Complementary Oligonucleotide—The ACCO GR20hh’

**Figure 3:**
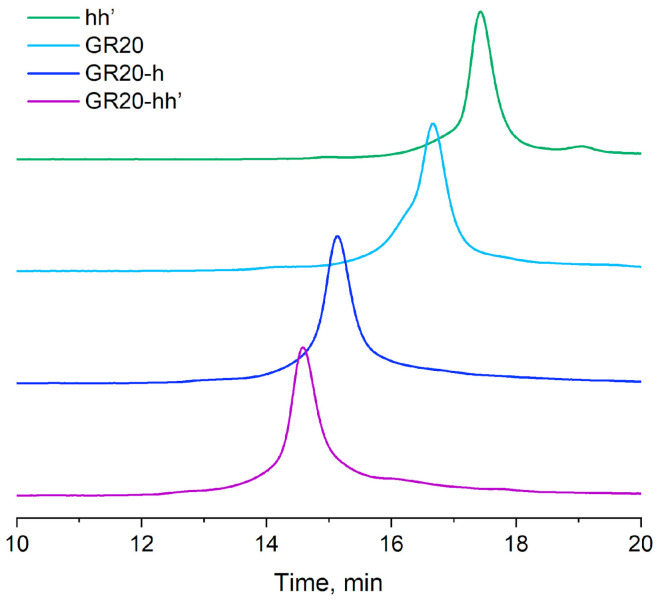
Size-exclusion HPLC: hh’ duplex (18 bp); the aptamer GR20 (46 nt); the aptamer GR20 with 20-mer 3′-extended oligonucleotide (GR20h, 66 nt); the ACCO GR20hh’ (66 nt + 18 nt).

To assess whether the hybridization of the aptamer GR20h with the oligonucleotide h’ was completed, an original version of the rapid gel filtration HPLC method was applied under conditions that minimize possible dissociation of the duplex in the ACCO GR20hh’ (Figure 3) [44].

The ACCO GR20hh’ is assembled completely (Figure 3).

**Figure 4:**
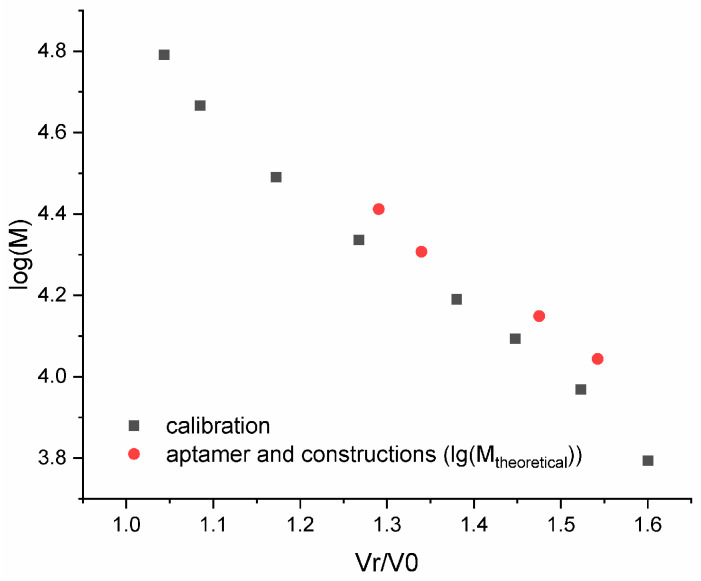
Dependence of the logarithm of the molar mass on the Normalized Retention Volume and a calibration curve (log10M = −1.541 vR/v0 + 6.337) for a set of DNA duplexes within a range of 10–100 base pairs (black) and the aptamers/ACCO (red).

The assembly of an ACCO is a key step in creating an aptamer construct. Usually, gel electrophoresis under non-denaturing conditions is used to evaluate the efficiency of ACCO assembly [45]; however, the non-covalent complex can dissociate in an electric field when the equilibrium is shifted. In addition, the method is not quantitative, since it involves the use of subsequent gel scanning. To minimize the dissociation of the complex and introduce a quantitative parameter such as UV absorption, special conditions for fast column gel filtration were developed [44]. Under these conditions, the 18-pair duplex itself does not dissociate, indicating complete assembly of the ACCO complex (Figure 3). A semi-logarithmic dependence of the molecular weight on the “retention” volume for the aptamers and the ACCO was calculated and compared with a similar dependence for the calibration duplexes (Figure 4). Only a minor deviation for the aptamer GR20h was found. The good correlation suggests that a putative secondary structure of the ACCO can be approximated as an extended imperfect duplex.

### 2.3. Thermal Stability of 18 Base Pair Duplexes Both Alone and as Part of the ACCO GR20hh’

**Figure 5:**
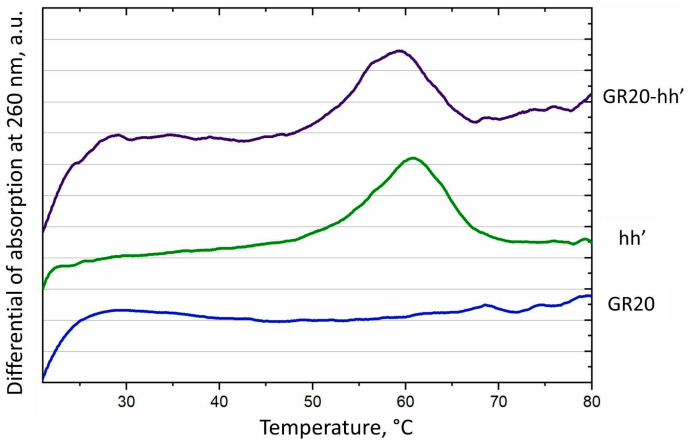
Differential of melting curves at 260 nm for the aptamer GR20 (blue), the duplex hh’ (green) and the ACCO GR20hh’ (purple).

Success in complete assembly of the ACCO GR20hh’ allowed the measurement of the thermal stability of the construct via standard UV melting at 260 nm (Figure 5). The aptamer GR20 itself did not show any characteristic hyperchromic effect upon heating, except for a slight increase in absorption at room temperature around 20–30 °C (Figure 5, blue line). In contrast, the ACCO GR20hh’ exhibited a characteristic curve upon melting: in addition to the initial rise noted for the aptamer GR20 itself, a peak with a melting temperature at 59 °C was observed on the differential curve, which, most likely, corresponds to the duplex (Figure 5, purple line). Indeed, the duplex hh’ alone melted characteristically with an experimental melting temperature of 61 °C, and the calculated melting temperature was 60 °C (Table 1). However, unlike the duplex hh’ alone, the duplex in the ACCO melted earlier by 2 °C and the melting was less cooperative (Figure 5, green line—hh’). Probably, the GR20 recognition module destabilizes the duplex in the construct, which should be taken into account during modular design.

### 2.4. Measuring Affinities of the Anti-EGFR DNA Aptamer GR20 and the ACCO GR20hh’ to the Recombinant Extracellular Domain of Human EGFR (EGFR*) Using Biolayer Interferometry (BLI)

Affinities of the aptamer GR20 and the ACCO GR20hh’ were measured via Biolayer Interferometry (BLI). The application of this technique for several anti-EGFR aptamers has already been described by us [36]. BLI measures interactions in real time; therefore, both the kinetics and equilibrium parameters can be calculated (Table 2). The kinetic data could stimulate speculations on the possible reasons for binding differences.

Consider the putative structure of the aptamer GR20 as a recognition part (Figure 1). Interactions of the aptamer GR20 with the EGFR* protein happen to be rather fast, but the resulting complex dissociates noticeably fast as well. The parent aptamer U31 interacts with and dissociates from EGFR* protein twice as slowly as the aptamer GR20. The imperfect terminal duplex of the aptamer U31 might restrict possible conformational fitting of the recognition part into EGFR* protein, which slows down the binding kinetics. On the other hand, it is possible that the imperfect terminal duplex of the aptamer U31 facilitates better anchoring on the protein, which, in turn, slows down the dissociation. On the contrary, the ACCO GR20hh’ interacts with EGFR* protein with about the same association rate as does the aptamer GR20, because the additional duplex hh’ does not fix the ends of the aptamer GR20 and does not restrict a possible conformational fitting into the EGFR* protein. The resulting complex dissociates slower than the aptamer GR20 because of the hh’ anchoring but faster than the aptamer U31, which has a more dynamic anchor (Table 2).

### 2.5. Interactions of the Aptamer GR20 and the ACCO GR20hh’ with Conventional GB Cell Line U87 and CCGBP 107

Next, direct interactions of the aptamer GR20 and the ACCO GR20hh’ with EGFR protein within the cell were measured via flow cytofluorometry using the standard EGFR+ GB cell line U87.

Previously, the fluorescent FAM label was introduced at the 5′-end of the aptamer to perform flow cytometry [46,47]. However, the specific structure of the ACCO GR20hh’ allows us to utilize a different approach: the FAM label is introduced not into the aptamer itself but at the 5′-end of the complementary oligonucleotide h’. The advantage of this approach is twofold: firstly, the presence of a signal on the cells will be an indicator of the interactions of the ACCO GR20hh’ with the cells, and, secondly, that would be an indicator of the stability of the ACCO GR20hh’ in the cellular environment during flow cytometry.

Conventional GB cell line U87 and cells of CCGBP 107 were tested. Cells of GB cell line U87 have a moderate relative amount of EGFR mRNA, close to the values for most CCGBP cells: 14.3 for U87 cell line versus 13.9 for cells of CCGBP 107 (Table 3).

**Figure 6:**
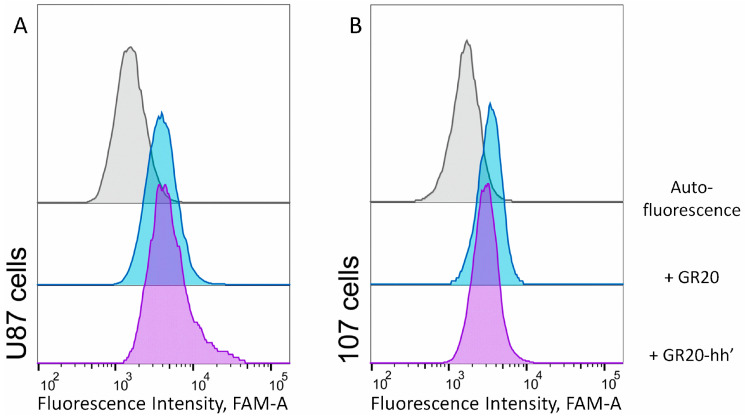
Flow cytometry data of direct interactions of (**A**) standard cell line U87 and (**B**) cells CCGBP 107 with 1 μM aptamer 5′-FAM-GR20 (blue) and the ACCO GR20hh’ with FAM-h’ (purple); room temperature, incubation for 30 min in the dark.

Mean Fluorescence Intensities for the aptamer GR20 and the ACCO GR20hh’ interacting with U87 and CCGBP 107 cells are practically identical (Figure 6). This opens the possibility to intercalate DOX into the ACCO GR20hh’ and test the complex on CCGBP 107 cells.

### 2.6. Binding of DOX to the Aptamer GR20 and the ACCO GR20hh’

**Figure 7:**
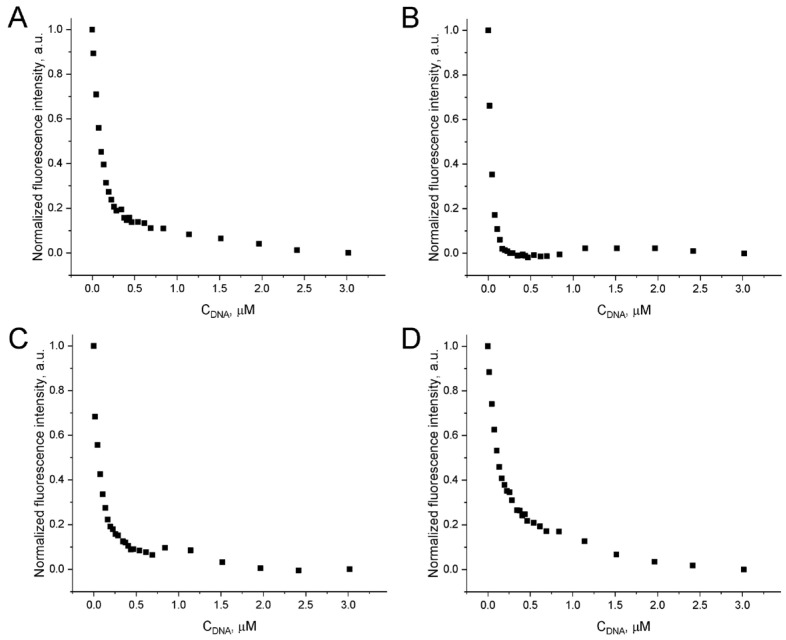
Quenching of fluorescence emission of 1.5 μM DOX at 530–550 nm (λ_ex_ = 460 nm) upon addition of the aptamer GR20 (**A**), the ACCO GR20hh’ (**B**), the aptamer U31 (**C**), and the double-stranded 18 bp duplex hh’ (**D**).

When intercalating DOX into the aptamer GR20 and the ACCO GR20hh’ is used to measure toxicity, the relative amounts/concentrations of DOX in the complex and free DOX are critical. The fluorescence of DOX is quenched when it intercalates into double-stranded regions of nucleic acids. To exclude the high background of free DOX while directly titrating the aptamer or the ACCO with an excess of DOX, backward titration was applied: the increasing amounts of the aptamer or the ACCO were added to a DOX solution with a fixed concentration. The resulting graph, showing the dependence of DOX fluorescence on the amount of the added aptamer/ACCO, represents a hyperbola (Figure 7). DOX binding is different for different aptamers/ACCO, showing different titration curvatures. Since the putative secondary structure of aptamers/constructs is a combination of defective hairpins, DOX binding occurs at several sites with different affinities, which makes it extremely difficult to build an adequate calculation model. In light of this, we selected the minimum distance of the hyperbola from the coordinate origin (Table 4) as a semi-quantitative curvature parameter.

The minimal basic aptamer GR20 binds DOX slightly better than the parent aptamer U31 and the isolated 18 bp duplex hh’ (Table 4). Combining the duplex with the aptamer GR20 to create the ACCO GR20hh’ generates a noticeable enhancement in binding. Evidently, DOX binds most efficiently to the ACCO GR20hh’, which has a double-stranded region. To estimate parameters of DOX binding to the aptamers and the ACCO, we applied formal approximation of DOX titration curves according to the Hill representation (Table 5). The aptamer GR20 binds DOX approximately 1.4-times better than the isolated 18 bp duplex hh’. At the same time, combining the duplex with the aptamer GR20 to create the ACCO GR20hh’ leads to a threefold improvement in binding (aK_d_ = 26 ± 1 nM) with a positive cooperativity coefficient of 1.6.

### 2.7. A Real-Time Analysis of the Kinetics of Uptake of the Complexes: DOX–Aptamers U31 and GR20 and DOX–ACCO GR20hh’, by Measuring the CI with xCELLigence Real-Time Cell Analysis (RTCA)usingh Cell Lines A-431, MCF-7, and Cells CCGBP 107

It is assumed that the aptamer/ACCO + DOX complexes bind to the EGFR receptors, internalize into the cell and, after releasing DOX, enter the nucleus and stop proliferation. At the same time, free DOX enters the cell from the medium via passive transmembrane diffusion. Both mechanisms will contribute to the kinetics of cell proliferation inhibition; however, only the rate of DOX entering the cytoplasm would differ. In the first case, this is the rate of endocytosis of the DOX + aptamer/ACCO + EGFR complex into the cell, entry into endosomes and release from endosomes into the cytoplasm. And, in the second case, it is the transmembrane diffusion of DOX.

**Figure 8:**
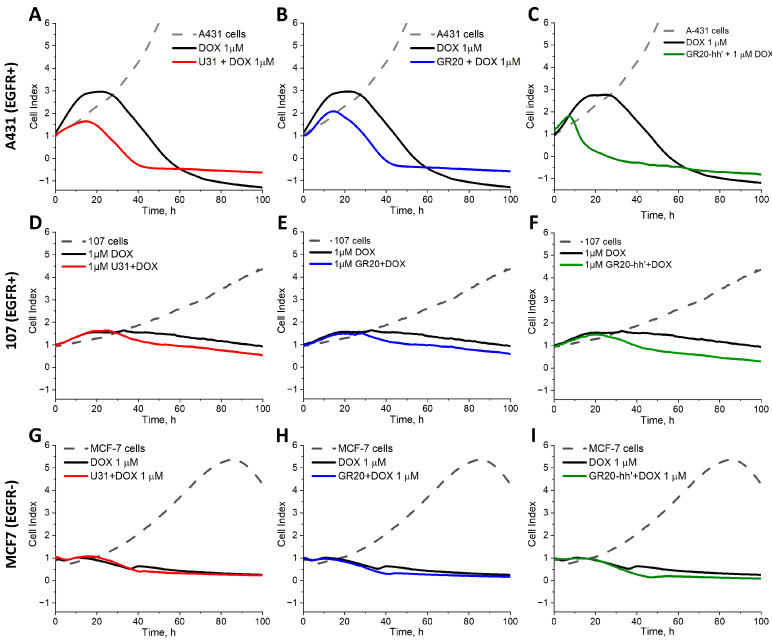
CI of A-431 (EGFR+++) (**A**–**C**), CCGBP 107 (EGFR+) (**D**–**F**) and MCF-7 (EGFR-) (**G**–**I**) cells incubated with free DOX (black), DOX in the complexes with aptamers U31 (red) (**A**,**D**,**G**), the GR20 (blue) (**B**,**E**,**H**), and the ACCO GR20hh’ (green) (**C**,**F**,**I**). The concentrations of the aptamers, the ACCO, and DOX are 1 μM.

For the first time, this study describes a method to monitor the possible difference in the kinetics of intracellular DOX internalization in real time for these two scenarios. The antiproliferative effect of free DOX and DOX within complexes with the aptamers U31 and the GR20, as well as with the ACCO GR20hh’, was monitored in real time by measuring CI, an arbitrary indicator provided by the xCELLigence device [48] (Figure 8 and Figure S1).

The device measures impedance—the resistance of alternating current in a chamber with cells, termed CI. The applied electrical potential does not affect the state and behavior of the cells [49]. The CI value is affected by the number of cells on the electrodes, i.e., the quality of cell interactions and the adhesive properties between each cell and the electrodes, as well as the size and shape of the cells. Thus, an increase or decrease in the CI value reflects a number of events: the growth, stretching, morphological changes, and, finally, death of the cells [49]. One of the most important advantages of the method is the lack of need to use labels to monitor cells in real time throughout the entire period of the experiment.

In some experiments, the IC50 value for 24 h incubation is completely different from that for 48 h incubation [50,51]. In this study, we recorded the change in the cell index (CI) for 100 h after adding free DOX or DOX complexes with oligonucleotides.

Noteworthily, despite the mentioned advantages of the method, its usage to assess the effect of aptamer complexes and conjugates on the cells has been sparsely described. A PubMed search showed only a single article published, by us, initially devoted to the study of the aptamers using the xCELLigence method [32].

Three types of cells were tested (Table 3). Conventional cell line A-431 with EGFR overexpression was taken as a positive control (accepted DB mRNA EGFR value 2978.0 nTPM, <https://www.proteinatlas.org accessed on 28 August 2025>); our experimentally measured value is 494 ± 5 arbitrary units. For CCGBP 107, our experimentally determined value for mRNA EGFR was 40-times lower, i.e., 13.8 ± 0.7 arbitrary units. MCF-7 cells have the lowest level of mRNA EGFR (accepted DB value 1.4 nTPM, <https://www.proteinatlas.org accessed on 28 August 2025>), i.e., approximately 2000-times less than for the cell line A-431, our experimentally estimated value was 3000-times less, 0.16 ± 0.03 arbitrary units. Therefore, MCF-7 cells were taken as a negative control.

Cell line A-431. Free DOX begins to stop the change in CI after 16 h and, after a small plateau, significantly reduces CI after 24 h of exposure to EGFR overexpressing cells A-431 (Figure 8). Complexes of DOX with the aptamers U31 and the GR20 reduce CI immediately, without a plateau, after 15–17 h, apparently because active endocytosis occurs faster than the free diffusion of DOX. At the same time, the DOX complex with the ACCO GR20hh’ reduces CI much earlier, after only 7 h, and the rate of decline in CI is much faster. It might occur due to a greater load of the ACCO GR20hh’ with DOX (Table 4). Absolute values for CI may slightly vary for different samples of the cell line A-431, but the general trend was the same.

Cells CCGBP 107. For the cells CCGBP 107, the trend of toxicity acceleration in DOX complexes is maintained, despite GB cells having 40-times less EGFR mRNA than the cell line A-431, but, as expected, the effect was less pronounced. The effect of free DOX shows a wide plateau with a conditional maximum at 34 h and then a slow decrease in the CI value. Complexes of DOX with the aptamers U31 and the GR20 did not show a plateau and decreased the CI after 23 h. The complex of DOX with the ACCO GR20hh’ showed a decrease in CI earlier, after only 21 h, and the rate of decrease was noticeably faster (Figure 8D–F). The character and kinetics of the decrease in CI were affected by a significant decrease in EGFR expression.

Cell line MCF-7. To test the hypothesis that the effect of complexes of DOX with aptamers and the ACCO is EGFR-dependent, experiments were carried out with a standard cell line MCF-7, which expresses only a small amount of EGFR mRNA (Table 3). None of the above-described effects were found. Insignificant differences in the behavior of free DOX and its complexes with the aptamers/ACCO were visible only for the 40 h time point, which most likely relates to the effect of DOX itself.

Therefore, the complexes of DOX with the anti-EGFR aptamers/ACCO GR20hh’ are internalized into EGFR+ GB cells faster than free DOX.

In the literature, a few studies have shown that complexes of DOX with aptamers are more toxic for target cells when static tests like MTT or MTS are used. Bagalkot, V et al. showed that free DOX has similar cytotoxicity on PSMA+ prostate cancer cells LNCaP and PSMA- cells PC3, whereas the cytotoxicity of the DOX–aptamer complex was significantly increased on the target LNCaP cells (cell viability: 52.8% ± 1.7 for LNCaP versus 75.2% ± 1.2 for PC3) [52].

Anti-EGFR-2 (HER2) aptamers have also been studied as “carriers” for DOX. Liu, Z et al. showed, by using the MTS test, that free DOX has similar cytotoxicity for breast cancer cells HER2+ SK-BR-3 and HER2- MDA-MB-231. Cell survival was about 60%, whereas the use of the DOX–aptamer complex enhances the specificity toward HER2+ cells, decreasing the level of SK-BR-3 cell survival down to 42% [53].

Using the XTT test, it was shown that the complex of DOX with the GMT-3 aptamer is more toxic for GB cell line A-172 (41.3% ± 3.8%) vs. the control cell line MCF-7 (82.1% ± 4.7%). At the same time, free DOX reduced the survival of both cell lines with equal efficiency (40.0% ± 6.3% for A-172 and 38.3% ± 2.9% for MCF-7) [54].

However, nonconventional conditions both for the DOX–aptamer interactions and cytotoxicity test do not allow for an estimation of the activity of the complexes.

In addition, there were no data on any effects of the DOX–aptamer complexes on the cell cultures from GB patients.

## 3. Materials and Methods

### 3.1. Aptamers and Oligonucleotides

DNA aptamers targeting EGFR and non-aptamer oligonucleotides were synthesized by GenTerra LCC. The aptamers and oligonucleotides used in this study were the following: U31 [5′-ATCCAGAGTGACGCAGCATTTGTTTAATATGTTTTTTAATTCCCCTTGTGGTGTGTTGTGGACACGGTGGCTTAGT-3′], GR20 [5′-ACGCACCATTTGTTTAATATGTTTTTTAATTCCCCTTGTGGTGTGT-3′], GR20h [5′-ACGCACCATTTGTTTAATATGTTTTTTAATTCCCCTTGTGGTGTGTTTCATTTAGGACCAACACAA-3′], h [5′-CATTTAGGACCAACACAA-3′], h’ [5′-TTGTGTTGGTCCTAAATG-3′].

For flow cytometry experiments, the GR20 aptamer and the h′ oligonucleotide with a fluorescent FAM label at the 5′-end were used. The aptamers were folded at a concentration of 1 μM in PBS containing 5 mM MgCl_2_ via denaturation at 95 °C for 5 min, slow cooling to room temperature.

### 3.2. Generation of the ACCO GR20hh’ and the 18-Bp Duplex hh’

The extended aptamer GR20h and the complementary oligonucleotide h’ were mixed in equal concentrations in the required buffer (PBS with 5 mM MgCl_2_ or Tris-HCl pH = 7.4 with the addition of 50 mM NaCl, 10 mM KCl, 5 mM MgCl_2_). To assemble the aptamer construct, the oligonucleotide mixture solution was heated for 5 min at 95 °C and then slowly cooled to room temperature. Similarly, the hh’ duplex was obtained by mixing two oligonucleotides: h and its complementary h’.

### 3.3. UV Spectroscopy

The melting curves of the oligonucleotide samples were recorded at a wavelength of 260 nm using a Hitachi U-2900 UV spectrophotometer (Hitachi, Tokyo, Japan) equipped with an SPR-10 thermoelectric controller. The optical path length was 10 mm. The temperature range used was 20–90 °C, with a heating rate of 0.5 °C/min [55].

### 3.4. Size-Exclusion HPLC

Chromatography of oligonucleotides was performed using an Agilent 1200 HPLC system (Agilent, Santa Clara, CA, USA) on a TSKgel G2000SWXL column (Tosoh Bioscience, South San Francisco, CA, USA). Column characteristics were as follows: length 30 cm, particle size 5 μm, average pore diameter 12.5 nm. Analysis conditions were as follows: temperature 25 °C, flow rate 0.5 mL/min, absorption detection at 260 nm. Preliminary column calibration was performed, as described previously [44]. Mobile phase composition was as follows: potassium phosphate buffer (60 mM KH_2_PO_4_ and 140 mM K_2_HPO_4_, pH 6.9) and acetonitrile in a ratio of 9:1 *v*/*v* [44].

### 3.5. Evaluation of DOX Intercalation into Aptamer via Fluorimetry Titration

Spectrofluorimetric titration was performed using a Nano-500 device (Allsheng, Hangzhou, China). Fluorescence intensity was recorded in a quartz cuvette with an optical path length of 1 cm. The following device settings were used: excitation wavelength—λ_ex_ = 490 nm; emission wavelength λ_em_ = 540 nm; spectral slit width—10 nm. Before each experiment, the baseline fluorescence of the buffer solution was recorded, which was then automatically subtracted by the device software. A stock solution of the anti-EGFR aptamers, the ACCO GR20hh’ or the 18-bp duplex hh’ was successively added to a 1.5 μM DOX in a Tris-HCl buffer. After mixing, the solution was kept for a minute to equilibrate. Fluorescence was recorded for each point three times. Calculations were performed based on the average fluorescence data.

### 3.6. Cell Cultivation

Human epidermoid carcinoma cell line A-431 (CRL-1555—ATCC), human GB cell line U87 (HTB-14™—ATCC), breast cancer adeno-carcinoma MCF-7 (HTB-22™—ATCC) were kindly provided by the Laboratory of Neurogenetics and Developmental Genetics of the Institute of Higher Nervous Activity and Neurophysiology of the Russian Academy of Sciences. Cells of the continuous culture isolated from the patient’s GB tumor tissue 107 were kindly provided by the Laboratory of Molecular Cellular Neurogenetics of the Burdenko Research Institute of Neurosurgery of the Ministry of Health of the Russian Federation. The cells were cultured in DMEM/F12 growth medium with sodium pyruvate supplemented with 10% fetal calf serum, 1% HEPES, 1% GlutaMAX and 1% streptomycin antibiotic in a 5% CO_2_ environment at 37 °C.

### 3.7. Monitoring of Interactions of Fluorescent Aptamers with Cells via Flow Cytometry

Cells were harvested at 80% confluency by adding 1 mL 0.25% trypsin-EDTA (PanEco Ltd., Moscow, Russia). After centrifugation at 1000× *g* for 3 min, cells were resuspended in PBS, 5 mM MgCl_2_ and counted. Then, cells were centrifuged and resuspended in growth medium with 10% FCS and 10^6^/mL incubated at 37 °C for 1 h to regenerate surface proteins. FAM oligonucleotide binding was analyzed by FC using 5 × 10^5^ cells U87 or 107 with solutions of the aptamer GR20 and the ACCO GR20hh’. The interaction with cells was carried out at 25 °C for 30 min with oligonucleotides at a concentration of 1 μM in 50% DPBS, 5 mM MgCl_2_ and 50% full-growth media. After 30 min of incubation in the dark, cells were washed three times with buffer, resuspended in 300 μL DPBS, 5 mM MgCl_2_ and analyzed. Fluorescence intensity was measured using CytoFlex (Beckman Coulter, Indianapolis, IN, USA). Further, 30,000 events were collected. Data were processed using FlowJo v10.6.2 (FlowJo LCC, Ashland, OR, USA).

### 3.8. Cell Viability Assay

To obtain a complex of the ACCO GR20hh’ with doxorubicin, DOX was incubated for 30 min at 25 °C in an equimolar amount with the folded ACCO GR20hh’ in PBS buffer with the addition of 5 mM MgCl_2_ final concentration of DOX, and the ACCO GR20hh’ in stock solution was 10 μM. Before addition to cells, stock solution was diluted 10-times with cell media.

CI was measured using xCelligence S16 cell analyzer (Agilent Technologies, Inc., Santa Clara, CA, USA). A-431, MCF-7, and 107 cells were uniformly seeded in the wells of 16-well E plates in duplicate. Then, the plate was placed in the analyzer inside an incubator (37 °C, 5% CO_2_) for continuous impedance recording. After 24 h, the test solutions were added to the cells: 1 μM DOX; 1 μM complexes of DOX with the anti-EGFR aptamers U31, GR20, and the ACCO GR20hh’ (the volar ratio DNA/DOX = 1:1). As a control, the changes in CI of cells incubated in the same volume of PBS buffer with 5 mM MgCl_2_ were recorded. After adding oligonucleotides to the cells, the CI was recorded for another 100 h. The data were processed using Origin 2021 software (OriginLab, Northampton, MA, USA).

## 4. Conclusions

In summary, we compared the anti-EGFR DNA aptamers U31 and GR20 and an aptamer construct with complementary oligonucleotide, ACCO GR20hh’. The ACCO GR20hh’ was assembled completely and showed stability both thermally and in the cell culture media. Affinities of the ACCO GR20hh’ to both recombinant extracellular domain EGFR and EGFR+ cells are comparable to the initial aptamer GR20. Intercalation of DOX into the aptamer GR20 and the ACCO GR20hh’ does not interfere with the affinities. Complexes of DOX with the aptamer GR20 and the ACCO GR20hh’ were internalized into the cells from GB patients more effectively than free DOX. The most effective internalization was observed with the ACCO GR20hh’.

Considering further development of the topic and extending the non-covalent complex frames by adding covalent conjugates, the xCELLigencet method will allow us to compare the kinetics of the activity of different aptamers, to compare kinetics of the activity of different payloads, to check correlations between affinities to the cells and toxicities of aptamer derivatives, to check correlations between internalization kinetics by CI and fluorescence microscopy, and many others.

## Figures and Tables

**Table 1:** Experimental and calculated melting temperatures of the duplex hh’, the original aptamer GR20 and the ACCO GR20hh’; 140 mM Na^+^, 10 mM K^+^, 5 mM Mg^2+^ (https://dna-utah.org/tm/tool.php accessed on 28 August 2025).

Name	Number of Pairs in the Proposed Structure (G-C Pairs)	Experimental Tm, °C	Calculated Tm, °C
GR20	4 + 5 + 2 (4)	20–30	
hh’	18 (7)	61	60
ACCO GR20hh’	4 + 5 + 2 + 18 (4 + 7)	59	

**Table 2:** Calculated parameters of the anti-EGFR DNA aptamers U31, GR20, and the ACCO GR20hh’ affinity to EGFR* protein.

Name	k_on_ × 10^−5^ (M × s)^−1^	k_off_ × 10^4^ s^−1^	K_D_, nM
U31	2.0 ± 0.2	29 ± 5	15.0 ± 1.7
GR20	4.3 ± 0.3	61 ± 2	14.2 ± 0.5
ACCO GR20hh’	3.7 ± 0.5	40 ± 2	10.8 ± 1.4

**Table 3:** A relative amount of EGFR mRNA in the cells according to RT-PCR data.

Cells	Experimental EGFR mRNA Amount (Relative Units)	Taken From DB EGFR mRNA Amount (nTPM) <https://www.proteinatlas.org accessed on 28 August 2025>
A-431	494 ± 5	2978.0
U87	14.3 ± 0.5	37.0
MCF-7	0.16 ± 0.03	1.4
CCGBP 107	13.8 ± 0.7	

**Table 4:** Binding parameters in arbitrary units as oligonucleotide concentrations (C_oligo_), representing minimal distances of titration hyperbolas from the origin. Approximate amount of DOX per molecule of the aptamer/construct.

Name	C_oligo_, a.u.	Approximate Amount of DOX per Molecule of Aptamer/Construct
GR20	0.41	7
U31	0.44	7
hh’	0.47	6
ACCO GR20hh’	0.17	11

**Table 5:** Apparent dissociation constants (aK_d_) and Hill coefficients (*n*) of DOX binding to the 18 bp duplex hh’, the aptamers U31 and the GR20, and the ACCO GR20hh’.

Name	aK_d_, nM	*n*
hh’	124 ± 3	0.96 ± 0.03
GR20	90 ± 3	1.13 ± 0.03
U31	47 ± 3	0.95 ± 0.04
ACCO GR20hh’	26 ± 1	1.58 ± 0.09

## Data Availability

The data presented in this study are available on request from the corresponding author.

## Supplementary Materials

The following supporting information can be downloaded at: https://www.mdpi.com/article/10.3390/ijms26178712/s1.

## Abbreviations

The following abbreviations are used in this manuscript:

A-431	Human epidermoid carcinoma cell line
ACCO	Aptamer Construct with Complementary Oligonucleotide
ADC	Antibody Drug Conjugate
ApDC	Aptamer Drug Conjugate
BLI	Biolayer Interferometry
CCGBP	Continuous Culture from GB Patient
CI	Cell Index
DB	Database
DOX	Doxorubicin
EGFR	Epidermal Growth Factor Receptor
EGFR*	recombinant extracellular domain of human EGFR
GB	Glioblastoma
mAb	Monoclonal Antibody
MCF-7	breast cancer adeno-carcinoma
MFI	Mean Fluorescence Intensities
MoREs	Molecular Recognition Elements
nTPM	Transcripts per million
RTCA	Real-Time Cell Analysis
U87	human glioblastoma cell line
