# Supplementary material for: Real-Time Kinetics of Internalization of Anti-EGFR DNA Aptamers and Aptamer Constructs into Cells Derived from Glioblastoma Patients as Indicated by Doxorubicin
Source: Int J Mol Sci. 2025 Sep 7;26(17):8712. doi: 10.3390/ijms26178712 (PMC12429564; doi:10.3390/ijms26178712)

Supplementary Figure S1. CI of cells A-431 (A-C), and MCF-7 (D-F) incubated with 1 μM free DOX (black), free aptamers U31 (red), GR20 (blue), and free ACCO GR20hh' (green).

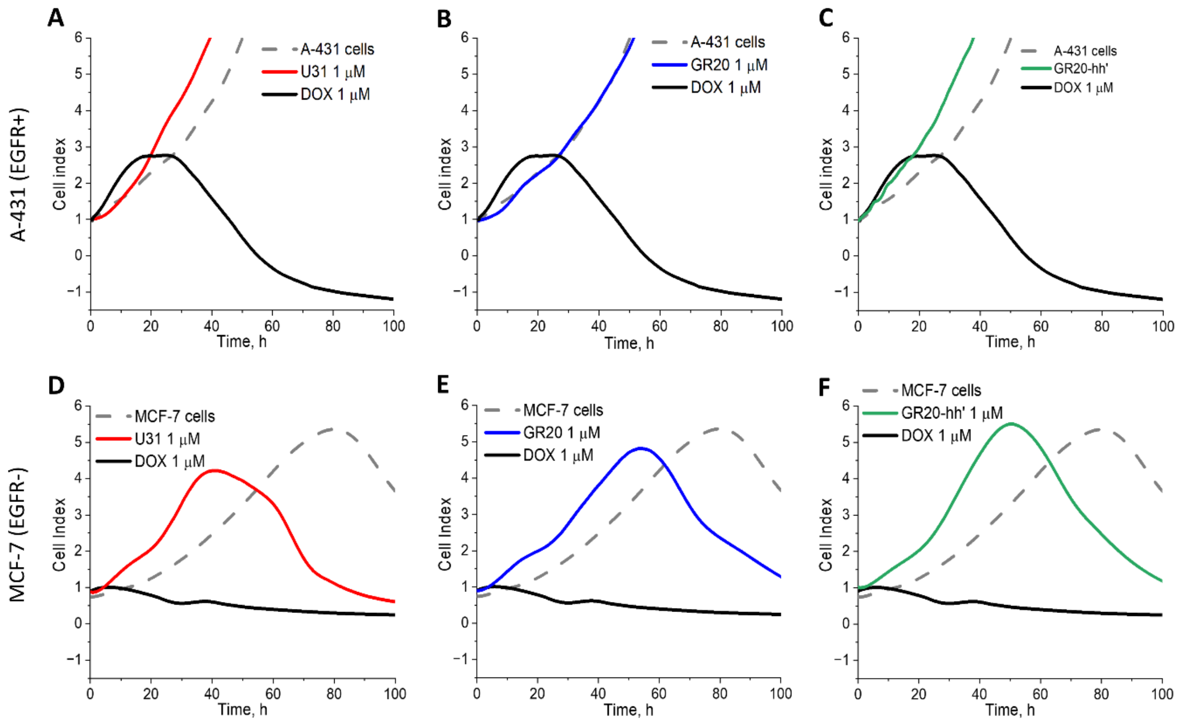

Supplement: Supplementary file 1 [file ijms-26-08712-s001.zip › ijms-3800530-supplementary.pdf]
